# Patterns of activity and body temperature of Aldabra giant tortoises in relation to environmental temperature

**DOI:** 10.1002/ece3.3766

**Published:** 2018-01-19

**Authors:** Wilfredo Falcón, Rich P. Baxter, Samuel Furrer, Martin Bauert, Jean‐Michel Hatt, Gabriela Schaepman‐Strub, Arpat Ozgul, Nancy Bunbury, Marcus Clauss, Dennis M. Hansen

**Affiliations:** ^1^ Department of Evolutionary Biology and Environmental Studies University of Zurich Zurich Switzerland; ^2^ Zurich Zoo Zurich Switzerland; ^3^ Clinic for Zoo Animals, Exotic Pets and Wildlife Vetsuisse Faculty University of Zurich Zurich Switzerland; ^4^ Seychelles Islands Foundation PO Box 853, Mahe Seychelles

**Keywords:** Aldabra, ectotherm, giant tortoise, Testudinidae, thermoregulation

## Abstract

We studied the temperature relations of wild and zoo Aldabra giant tortoises (*Aldabrachelys gigantea*) focusing on (1) the relationship between environmental temperature and tortoise activity patterns (*n* = 8 wild individuals) and (2) on tortoise body temperature fluctuations, including how their core and external body temperatures vary in relation to different environmental temperature ranges (seasons; *n* = 4 wild and *n* = 5 zoo individuals). In addition, we surveyed the literature to review the effect of body mass on core body temperature range in relation to environmental temperature in the Testudinidae. Diurnal activity of tortoises was bimodally distributed and influenced by environmental temperature and season. The mean air temperature at which activity is maximized was 27.9°C, with a range of 25.8–31.7°C. Furthermore, air temperature explained changes in the core body temperature better than did mass, and only during the coldest trial, did tortoises with higher mass show more stable temperatures. Our results, together with the overall Testudinidae overview, suggest that, once variation in environmental temperature has been taken into account, there is little effect of mass on the temperature stability of tortoises. Moreover, the presence of thermal inertia in an individual tortoise depends on the environmental temperatures, and we found no evidence for inertial homeothermy. Finally, patterns of core and external body temperatures in comparison with environmental temperatures suggest that Aldabra giant tortoises act as mixed conformer–regulators. Our study provides a baseline to manage the thermal environment of wild and rewilded populations of an important island ecosystem engineer species in an era of climate change.

## 1 INTRODUCTION

Activity and body temperature of reptiles depend on the external thermal fluctuations in the environment and are both drivers and consequences of their physiological and behavioral biology, which ultimately affects their ecology (Heatwole, 1976; Huey & Stevenson, 1979; Lailvaux & Irschick, 2007; Van Damme, Bauwens, & Verheyen, 1991). Many physical processes can affect the thermal environment of reptiles, including for example fluxes of radiative heat, convection, conduction, and wind (Cossins & Bowler, 1987; Willmer, Stone, & Johnston, 2005). However, understanding this complex thermal environment alone does not allow an adequate description of a reptiles’ activity patterns and core body temperature (*T*_bc_; Table 1 lists the terms adopted here and their definitions). Rather than being thermally passive, that is, with a body temperature driven only by fluctuations in environmental temperature, many reptiles have been shown to exhibit complex thermoregulatory behaviors and physiological processes to maintain their *T*_bc_ within a narrow range, albeit within limits determined by environmental conditions (Paladino, O'Connor, & Spotila, 1990; Seebacher & Franklin, 2001; Slip & Shine, 1988).

**Table 1:** Terms related to thermoregulation ecology used in this article

Term	Definition
*T*_a_	Available environmental temperature envelope (e.g., available *T*_air_ and *T*_sun_ in our case) for temperature regulation (e.g., throughout a given time period or study; °C)
*T*_a mean_	Mean environmental temperature (from *T*_air_ and *T*_sun_; °C)
*T*_air_	Temperature recorded by loggers placed in a shaded area used by tortoises (equivalent to air temperature); and air temperature reported in studies for the review section; °C
∆*T*_air_	Environmental temperature range given by the minimum and maximum temperatures recorded over a certain period from loggers placed in the shade (range in °C)
*T*_a‐opt_	Optimum mean air temperature at which activity is maximized (°C)
*T*_a‐opt range_	Optimum air temperature range given by the minimum and maximum temperatures at which the active state surpasses the inactive state of tortoises (°C)
*T*_bc_	Core body temperature (i.e., gut temperature; °C)
∆*T*_bc_	Core body temperature range given by the minimum and maximum temperatures recorded over a certain period (˚C)
*T*_be_	External (contact) body temperature (i.e., carapace, extremities and skin fold; °C)
*T*_sun_	Temperature recorded by loggers placed in a sun‐exposed area used by tortoises (includes radiative temperature; °C)

Definitions based and modified from Cossins and Bowler (1987), Blatteis et al. (2001) and Willmer et al. (2005).

For example, when basking in sunny places, the common Puerto Rican ameiva (*Ameiva exsul*) can attain a *T*_bc_ that is higher than the air temperature (*T*_air_), which allows them to be active later while foraging in the shade (Rivera‐Vélez & Lewis, 1994). In addition, salt water crocodiles (*Crocodylus porosus*) can employ shuttling behavior between water and land to regulate their *T*_bc_ (Seebacher, Grigg, & Beard, 1999). On the other hand, eastern bearded dragons (*Amphibolurus barbatus*) can exert physiological reactions of metabolism and circulation in response to varying *T*_a_ to control heating and cooling rates and maintain preferred *T*_bc_ (Bartholomew & Tucker, 1963). Furthermore, green iguanas (*Iguana iguana*) can display physiologically generated circadian rhythms in a constant environmental temperature, similar to those recorded in endotherms (Tosini & Menaker, 1995).

Another factor considered important in influencing fluctuations of *T*_bc_ in reptiles is their body size, as the surface‐to‐volume ratio influences the rate at which heat is exchanged with the environment. Hence, due to a relatively small surface‐to‐volume ratio for larger reptiles, their *T*_bc_ is expected to be less responsive to the thermal environment than that of smaller ones (i.e., more stable; Zimmerman & Tracy, 1989). Body size also affects the rate of heat absorption from the sun (Brattstrom, 1965), as larger bodies have a higher surface area, which in part drives their external body temperature (*T*_be_). McNab and Auffenberg (1976) suggested that larger reptiles have a relatively low thermal conductance because their small surface‐to‐volume ratio and thick integuments could give them a substantial heat storage capacity, and because larger reptiles take longer to attain thermal equilibrium with the environment. For example, larger salt water crocodiles are able to attain not only higher but also more stable *T*_bc_ than smaller ones, at least in part due to thermal inertia (Seebacher et al., 1999).

Here, we describe activity and body temperature fluctuations of Aldabra giant tortoises (*Aldabrachelys gigantea* Schweigger 1812) in both their natural habitat and in captivity. Specifically, we focus on (1) the relationship between environmental temperature (*T*_a_) and activity patterns of wild tortoises to determine their optimal environmental temperature range (*T*_a‐opt_); and (2) the body temperature fluctuations of captive and wild tortoises, including how their core and external body temperatures vary in relation to environmental temperatures, and whether body mass influences the response of tortoise core body temperatures to environmental temperatures. In addition, we surveyed the literature to investigate the effect of body mass on the body temperature ranges of Testudinidae in relation to air temperature.

## 2 MATERIALS AND METHODS

### 2.1 Study species and study sites

The Aldabra giant tortoise is endemic to Aldabra Atoll, Seychelles, with a stable population of an estimated 100,000 tortoises (Bourn et al., 1999; Turnbull et al., 2015). We studied wild tortoises on Picard Island, Aldabra, as well as captive ones in the Masoala Rainforest exhibit at Zurich Zoo, Switzerland. Aldabra is a raised coral atoll in the Western Indian Ocean and has a tropical climate with a wet season (November to April) and a dry season (May to October). The timing and distribution of rainfall vary greatly from year to year, which is unevenly distributed across the atoll, and directly drive spatiotemporal patterns in vegetation productivity (Haverkamp et al., 2017; Shekeine et al., 2015). In Zurich Zoo, the Masoala Rainforest exhibit is an 11,000‐m^2^ greenhouse ecosystem covered with translucent foil permitting 75% of daylight and 50% of UV radiation to enter and has an artificial rain and fog systems and an air‐circulating heating system with the aims of maintaining minimum temperatures of 24° during the day and 18°C during the night (Bauert, Furrer, Zingg, & Steinmetz, 2007).

### 2.2 Environmental temperatures and tortoise activity patterns

The environmental temperature range at which activities such as locomotion, feeding, and mating occur in tortoises is narrow (Meek, 1984). As other reptiles, tortoises have to select environmental temperatures that allow them to maintain *T*_bc_s at which manifold processes are optimized to increase their fitness, for example physiological, locomotory, and foraging ones. We examined activity patterns of wild tortoises in relation to air temperature (*T*_air_) to derive an approximation for the optimal environmental temperature at which activity is maximized (*T*_a‐opt_). We derived activity based on accelerometer (ACC) data obtained from data loggers mounted on eight tortoises (06:00–24:00) for 2 years, as part of an ongoing long‐term movement ecology study (we assumed no activity between 00:00 and 06:00). ACC data were recorded every 5 min for a burst of 5‐s, during which 36 voltage readings were recorded. To assign a state of either active (1) or inactive (0) for/within each 5‐min period, a rolling mean of the standard error (*SE*) of the ACC bursts was used to capture fluctuations in the ACC waveform. We set the threshold of the rolling average *SE* to five, to create binary data (similar to Nielsen, Pedersen, Herskin, & Munksgaard, 2010). The activity data were then coupled with *T*_air_ obtained from the weather station located at the research station on Picard, which was collected every 15 min.

### 2.3 Body temperature of *Aldabrachelys gigantea*

We studied body temperature fluctuations in five captive tortoises from Zurich Zoo and four wild tortoises from Aldabra. The zoo tortoises were housed in a compartment within the Masoala Rainforest exhibit, while the wild tortoises, selected for their different body masses (Table 2), were temporarily housed in two 36 m^2^ enclosures located on Picard. Data from the captive tortoises were obtained during summer 2009 (ZRH summer; *n* = 3) and winter 2010 (ZRH winter; *n* = 5), while data from the wild tortoises were obtained during April 2014 (Aldabra; *n* = 4). In the course of the trials, all animals had ad libitum access to drinking water, food and access to both shade and sunlight. Food consisted of hay, freshly cut grass, and vegetables for the captive tortoises, and freshly cut leaves from native woody vegetation of known food species for the wild tortoises.

**Table 2:** Summary statistics of the environmental (*T*_air_ and *T*_sun_) and core body temperatures (*T*_bc_) of Aldabra giant tortoises (*Aldabrabrachelys gigantea*), with different body mass, exposed to different thermal environments. Temperatures are indicated in degrees celsius, and the acrophase is indicated in hours. The mean, maximum, and minimum temperatures refer to the overall temperatures during the trials, while the range refers to the mean daily temperature range

Environmental temperature	Study	Mean	±*SD*	Min	Max	Daily range	Acrophase
Sun	ZRH Winter	18.0	3.0	14.4	32.1	10.3	14.0
Air (shade)	ZRH Winter	16.5	2.1	13.9	23.7	6.4	14.9
Mean	ZRH Winter	17.2	2.5	14.2	27.4	8.2	14.4
Sun	ZRH Summer	25.4	5.4	18.2	36.6	15.3	14.5
Air (shade)	ZRH Summer	22.0	3.1	17.1	28.6	8.7	15.3
Mean	ZRH Summer	23.7	4.2	17.7	32.6	11.8	14.7
Sun	Aldabra	31.6	7.3	23.0	56.5	18.5	14.0
Air (shade)	Aldabra	29.1	2.5	24.0	38.5	6.5	15.9
Mean	Aldabra	30.3	4.4	23.8	43.8	12.4	14.5

Tortoise	Mass (kg)	Study	Mean	±*SD*	Min	Max	Daily range	Acrophase
JVS	14	ZRH Winter	20.8	1.6	17.0	26.0	3.1	19.04
JVL	19	ZRH Winter	21.5	1.3	18.6	25.5	2.7	20.26
HMA	100	ZRH Winter	22.2	0.9	20.1	24.6	1.4	20.98
SBY	140	ZRH Winter	21.1	1.0	19.2	23.9	1.6	20.28
BBY	180	ZRH Winter	20.5	0.8	18.4	23.0	1.5	21.53
HMA	100	ZRH Summer	29.9	2.1	25.1	34.1	5.0	19.63
SBY	140	ZRH Summer	30.1	2.4	24.7	34.2	5.7	20.03
BBY	180	ZRH Summer	29.7	2.0	24.2	33.2	4.7	20.57
BEL	39	Aldabra	29.9	1.6	26.0	34.5	4.7	18.13
UNM	48	Aldabra	29.9	1.7	26.0	34.0	4.9	17.86
CFK	61	Aldabra	30.2	1.6	26.5	35.0	4.4	18.34
LDX	97	Aldabra	31.0	1.7	26.0	34.5	5.0	17.86

Environmental temperatures were recorded with temperature loggers every 15 min for all study periods. In Zurich Zoo, we used 11‐bit Thermocron HC temperature loggers (±0.06°C accuracy; OnSolution Pty Ltd., NSW, Australia), while on Aldabra, we used iButton^®^ temperature loggers (±0.05°C accuracy; Maxim Integrated, San Jose, CA, USA). In both sites, loggers were placed at two locations (shaded and directly exposed to sunlight) at a height of 0.3–0.5 m. Surface (external) body temperatures (*T*_be_) were measured using infrared temperature pistols: *Raytek Fluke 566* in Zurich Zoo (0.01°C accuracy; Raytek Corporation, Santa Cruz, USA) and *testo 810* on Aldabra (0.1°C accuracy; Testo SA & Co., Mönchaltorf, Switzerland). To test whether logger and pistol data could be reliably compared, logger temperatures were directly measured using the infrared temperature pistol in Zurich Zoo, and data were highly correlated (*T*_air_: *z *=* *150.68, *p *<* *.001, Kendall τ = 0.89; *T*_sun_: *z *=* *84.05, *p *<* *.001, Kendall τ = 0.89). We fed temperature loggers to the tortoises and recorded their internal temperature (*T*_bc_) every 15 min. Feces were examined daily for the loggers, which were voided 10–20 days after ingestion. In Zurich Zoo, three tortoises (100–180 kg; see Table 2 for individual body masses) were fed the data loggers during the summer trial and five tortoises for the winter trial (14–180 kg; same three individuals as in summer, plus two additional ones). On Aldabra, four tortoises (39–97 kg) were monitored. We additionally measured the following temperatures at 1–3‐hr intervals for a 48–72‐hr period, commencing 5 days after feeding the loggers to the tortoises: (1) surface of the carapace (the center of each of the 13 main scutes, vertebral and costal), (2) the four extremities (each extremity in the region of the metacarpal/metatarsal joints), and (3) the deep skin folds (the skin at the deepest point underneath the carapace between each fore extremity and the neck, and next to each hind extremity; i.e., four measurements).

### 2.4 Body size and temperature in testudinidae

To investigate thermal inertia, and inertial homeothermy, we collated data on ∆*T*_bc_, ∆*T*_air_, and on body mass in Testudinidae from the scientific literature. We searched the literature and selected studies that presented the aforementioned data for at least one Testudinid species, or summarized mean values for a group of individuals. When studies only showed results graphically, we extracted the data from figures using WebPlotDigitizer v. 3.10 (Rohatgi, 2017; http://arohatgi.info/WebPlotDigitizer). We then assessed whether ∆*T*_air_ and/or *mass* significantly influenced ∆*T*_bc_ using correlation analyses. We restricted our assessment to Testudinidae, rather than Testudines, as oceanic/aquatic and semiaquatic species are subject to different physical processes than terrestrial species (i.e., dissimilarities in heat dissipation caused by differences in convection and conduction properties of air and water).

### 2.5 Statistical analyses

We performed all statistical analyses using R v. 3.3.0 (R Core Team, 2016) and report mean values and standard deviations (±*SD*). When plotting data, we fitted nonparametric locally weighted regressions using the nearest neighbor approach (loess; with *t*‐based approximation 95% CI), using the package “ggplot2” (Wickham, 2016). We determined the temperature range at which tortoises maximize their activity (*T*_a‐opt_) by performing kernel density estimation. We partitioned the activity data in active and inactive states at a given temperature, which yielded a relative density distribution for each state with the area under the curve of the probability distributions adding to one. We visualized the kernel density estimation by partitioning the active and inactive states using R package “ggplot2” and expected the distribution of the activity probability in response to environmental temperature to show a bimodal distribution for the inactive state, with the active state exhibiting greater levels of activity in between. In addition to calculating the *T*_bc_ ranges of the Aldabra giant tortoises and that of *T*_air_ and *T*_sun_, we also fitted cosines of the angles to the observed *T*_bc_ data with circular–linear regression (e.g., Jammalamadaka & Lund, 2006; Kinahan, Inge‐moller, Bateman, Kotze, & Scantlebury, 2007) using the R package “psych” (Revelle, 2016) and calculated the acrophase (time period during which the peak of *T*_bc_ occurs).

We used the package “lme4” (Bates, Machler, Bolker, & Walker, 2015) to construct generalized linear mixed effects models with random factors (GLMMs) following Zuur, Ieno, Walker, Saveliev, and Smith (2009). For the activity data, we tested the effects of *year*,* time*,* season* (wet and dry), *T*_air_, and the interactions between *time* and *season*, and *T*_*air*_ and *season* on tortoise *activity* (active or inactive state; using logistic regression analysis with binomial family and link “log”; from here on “activity model”). We added *individual* (tortoise) and *day* (date) as random factors to account for individual variation and repeated measures. The analysis was limited to 06:00–20:00, and the wet season comprised the months November–April and the dry season May–October. To account for the nonlinear relationship between *activity* and *time*, we discretised continuous time into four periods (I–IV), following the overall activity turning points through time, and comprising 06:00–08:00, 08:15–13:30, 13:45–17:30, and 17:45–20:00, respectively (see S1 in Supplementary Information). Furthermore, to assess the factors that influence *T*_bc_ of giant tortoises during our trials, we tested the following explanatory variables: *trial*,* time*,* T*_air_, *mass,* and the interaction between *T*_air_ and *mass* (from here on “thermoregulation model”). We included a random factor with individual tortoises interacting with *trial* and a random factor with day (date) to account for individual variation and repeated measures among trials. Because they are correlated, *T*_air_ was selected over *T*_sun_ and *T*_a mean_ as a explanatory variable based on model selection (∆AIC). Moreover, and similar to the activity model, we discretised continuous time into three periods (I–III) following the turning points of *T*_bc_ through time for each independent trial and comprising the morning period when tortoises are cooling down, the morning–afternoon period when tortoises are heating up, and the night period when tortoises start to cool down, respectively, to account for the nonlinear relationship between *T*_bc_ and *time* (see S2 in Supplementary Information). We obtained *p*‐values for the explanatory variables using the Satterthwaite degrees of freedom approximation (implemented in package “lmerTest” for the thermoregulation model; Kuznetsova, Brockhoff, & Christensen, 2016).

## 3 RESULTS

### 3.1 Environmental temperatures and tortoise activity patterns

**Figure 1:**
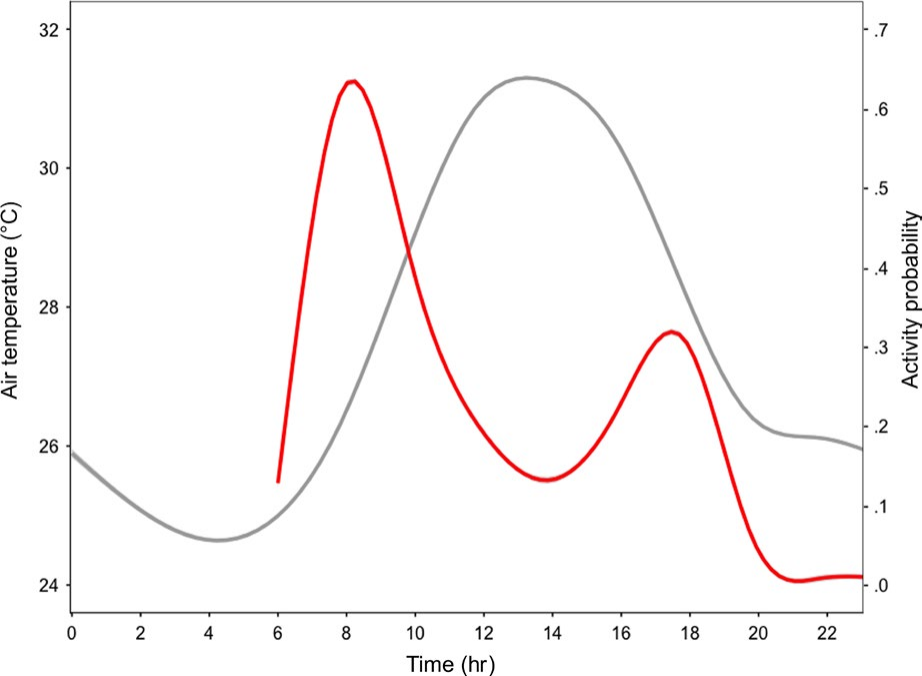
Bimodal distribution of the probability of activity (red line) of wild Aldabra giant tortoises (*Aldabrachelys gigantea*) and environmental temperature (gray line) per hr/day. Line smoothing by local regression loess

**Figure 2:**
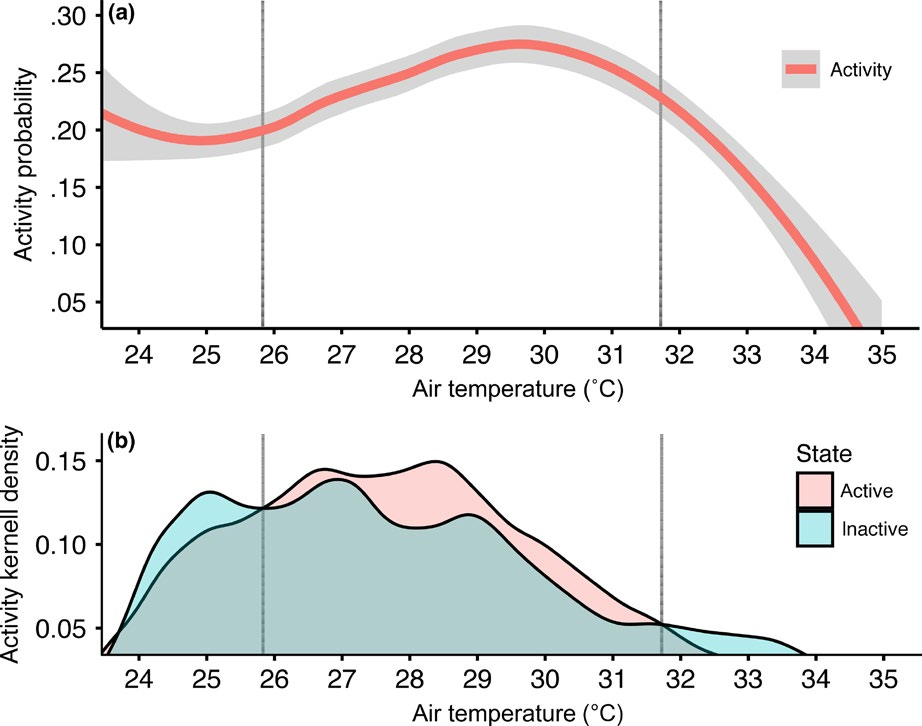
Probability of Aldabra giant tortoise (*Aldabrachelys gigantea*) activity as a response to air temperature (*T*_air_) (a) and kernel density plot of active and inactive states for a given *T*_air_ (b). Line smoothing by local regression loess (shading indicates the 95% CI). Vertical lines delimit the temperature range at which activity is maximised. *T*_air_ was recorded from Picard Island weather station daily along with tortoise activity data from eight individuals during 2013–2014

Diurnal activity of Aldabra giant tortoises on Aldabra was bimodally distributed, with the highest activity levels occurring during the morning, and during the late afternoon, albeit at comparatively lower levels (Figure 1). The mean *T*_air_ during the active state was 27.9°C (±2.6; 25%–75% quartile = 26.0–29.6°C). Notably, the probability of activity rapidly decreased as the environmental temperature increased above ca. 31–32°C (Figure 2a). Moreover, the kernel frequency distribution of the active state highlights that the temperature range of the active state in tortoises is 25.8–31.7°C (Figure 2b). In the activity model, the activity patterns of tortoises were significantly influenced by *T*_air_, as well as *time*,* season,* and the interactions between *T*_air_ and *time*, and *T*_air_ and *season* (*p *<* *.001), but not by year (*p *=* *.87; see S3 in Supplementary Information for model statistics and S4 for seasonality plot).

### 3.2 Body temperature of *Aldabrachelys gigantea*

**Figure 3:**
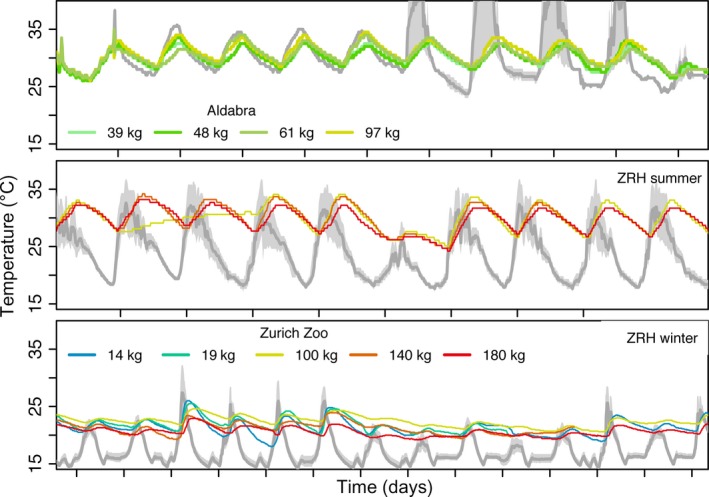
Temporal fluctuations in the environmental temperature range (*T*_air_ and *T*_sun_; gray fill) and core temperature recorded by data loggers in the gastrointestinal tract of Aldabra giant tortoises (*Aldabrachelys gigantea*) in different trials. The dark gray line depicts *T*_*a* mean_, and cool to warm colored lines depict ascending mass range for individual tortoises (with mass given in the legend) One trial was performed on Aldabra Atoll with wild tortoises, and two trials were performed at Zurich Zoo (during winter and summer). Tick marks in the *x*‐axis depict a 24‐hr interval: peaks of environmental temperature indicate temperature at ~mid‐day, and lowest point of the valleys indicates temperatures at ~midnight

Temperatures measured in shaded and sunny areas in each of the trials had similar daily minima but different maxima (Table 2), with considerable day‐to‐day variation in all trials (Figure 3). On Aldabra, we recorded relatively higher temperatures, in some cases above 40°C, in the sunny areas. The general daily patterns of *T*_bc_ lagged behind those of *T*_a_. Tortoises on Aldabra seemed to behave as thermoconformers at the beginning of the trial (first 5 days, where the *T*_bc_ of tortoises followed *T*_a_ closely). At the beginning of the ZRH summer trial, the 100‐kg individual seems to have avoided basking in the sun. During the trials, there were some perturbations in the *T*_a_ cycles, and the ability of tortoises to cope with these depended on the direction of the perturbation. Tortoises were able to maintain a stable *T*_bc_ when *T*_a_ increased above normal levels (e.g., Aldabra) but had difficulties doing so when *T*_a_ decreased sharply (e.g., ZRH summer and the last day at Aldabra). Overall, tortoises were able to maintain their *T*_bc_ above low mean *T*_a_ and below high mean *T*_a_. Tortoises maintained their mean *T*_bc_ at 30.1°C ± 1.9 during the ZRH summer and Aldabra trials (for all tortoises combined, *n* = 7; Table 2). In contrast, during the ZRH winter trial, tortoises maintained a mean *T*_bc_ of 21.2°C ± 1.3, albeit higher than mean *T*_a_. The mean daily *T*_bc_ of tortoises varied by 3.7°C ± 0.9 on Aldabra, 4.7°C ± 1.2 during ZRH summer, and 4.9°C ± 1.0 during ZRH winter. Moreover, only during the winter trial, where juveniles were included, did we observe that the rate of heat loss during the night increased as mass decreased (Figure 3). On the other hand, only on Aldabra when *T*_a_ was above 25°C did the *T*_bc_ of tortoises reach values very close to the minimum *T*_a_.

**Figure 4:**
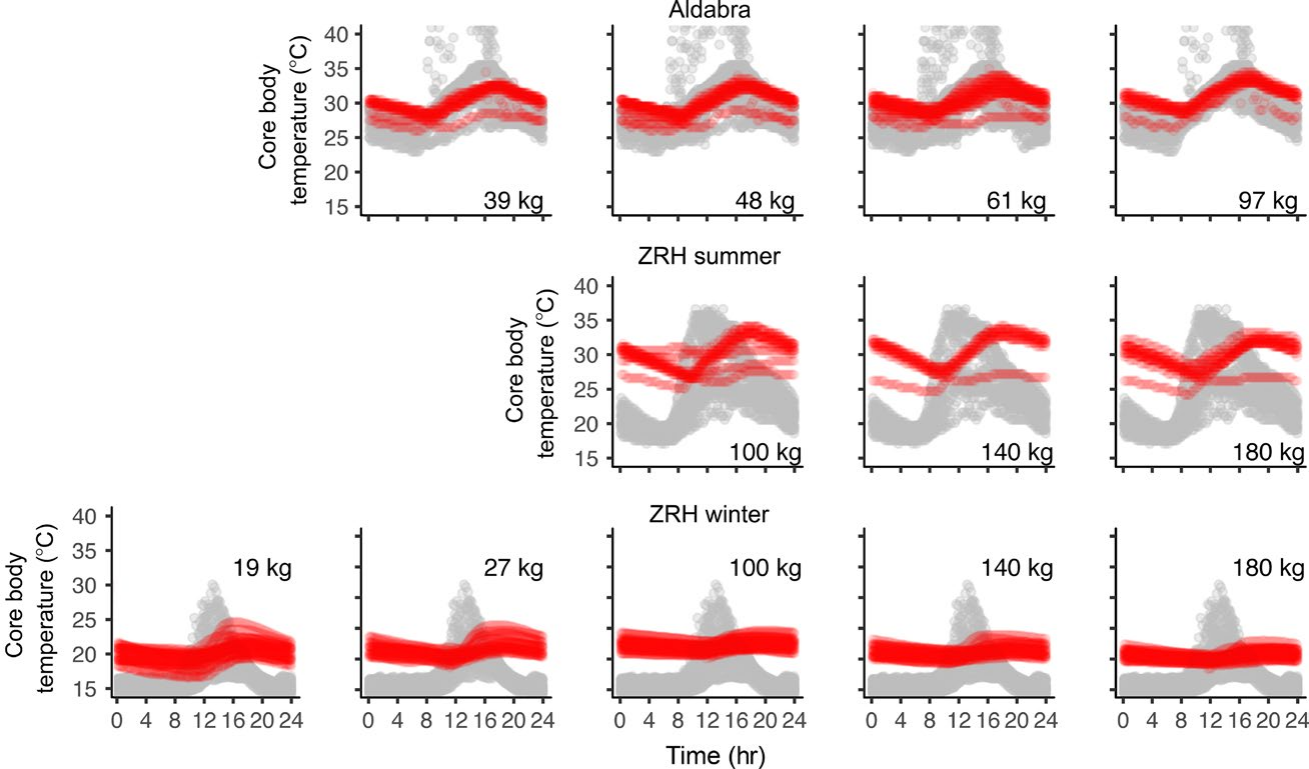
Daily environmental temperatures (gray; *T*_air_ and *T*_sun_) and core body temperature (red; *T*_*bc*_) of Aldabra giant tortoises (*Aldabrachelys gigantea*) of different mass across different environmental temperature ranges (trials)

Viewing the aggregated variability in the tortoises’ *T*_bc_ over a 24‐hr period, the variable dependence of the effects of mass on *T*_a_ (due to different *T*_a_ ranges available) and their influence on *T*_bc_ became clear (Figure 4). During winter, the magnitude of the response of *T*_bc_ to daily changes in *T*_a_ decreased with size, and only the smallest tortoises were able to briefly reach *T*_bc_ close to *T*_a‐opt_. However, as *T*_a mean_ reached values and temperature ranges closer to *T*_a‐opt_, larger tortoises were able to increase and maintain their mean *T*_bc_ close to the upper range of *T*_a‐opt_ (i.e., ZRH summer and Aldabra trials). In general, as *T*_a mean_ and minimum *T*_a_ increased, so did the *T*_bc_ of the tortoises (Table 2), but most of *T*_bc_ readings remained above *T*_a mean_.

The magnitude of daily ∆*T*_bc_ differed among trials (Table 2). Overall, there was a positive correlation between the daily ∆*T*_bc_ and ∆*T*_air_, but it was only significant for the ZRH trials (*z *=* *5.87 and 4.12, *p *<* *.01, *Kendall* τ = 0.43 and 0.55 for the winter and summer trials, respectively). On Aldabra, there was no significant correlation (*z *=* *1.46, *p *=* *.14, *Kendall* τ = 0.16). For ZRH winter, ∆*T*_bc_ decreased from the smallest to the largest animal (*z *=* *−4.76, *p *<* *.001, *Kendall* τ = −0.37). In contrast, ∆*T*_bc_ of tortoises during ZRH summer (without smaller, juvenile tortoises) and on Aldabra remained virtually the same between individuals of different mass (*z *=* *−0.73 and 0.28, *p *=* *.47 and *p *=* *.78, Kendall τ = −0.10 and 0.03, respectively). Moreover, in ZRH winter, the ∆*T*_bc_/∆*T*_air_ quotient was negatively correlated with tortoise body mass (*z *=* *−5.87, *p *<* *.001, *Kendall* τ = −0.45). However, we found no correlation in the ZRH summer trial (where no juveniles were included) or the Aldabra trial (*z *=* *−0.71 and 0.12, *p *=* *.48 and 0.91, Kendall τ = −0.10 and 0.01, respectively).

**Figure 5:**
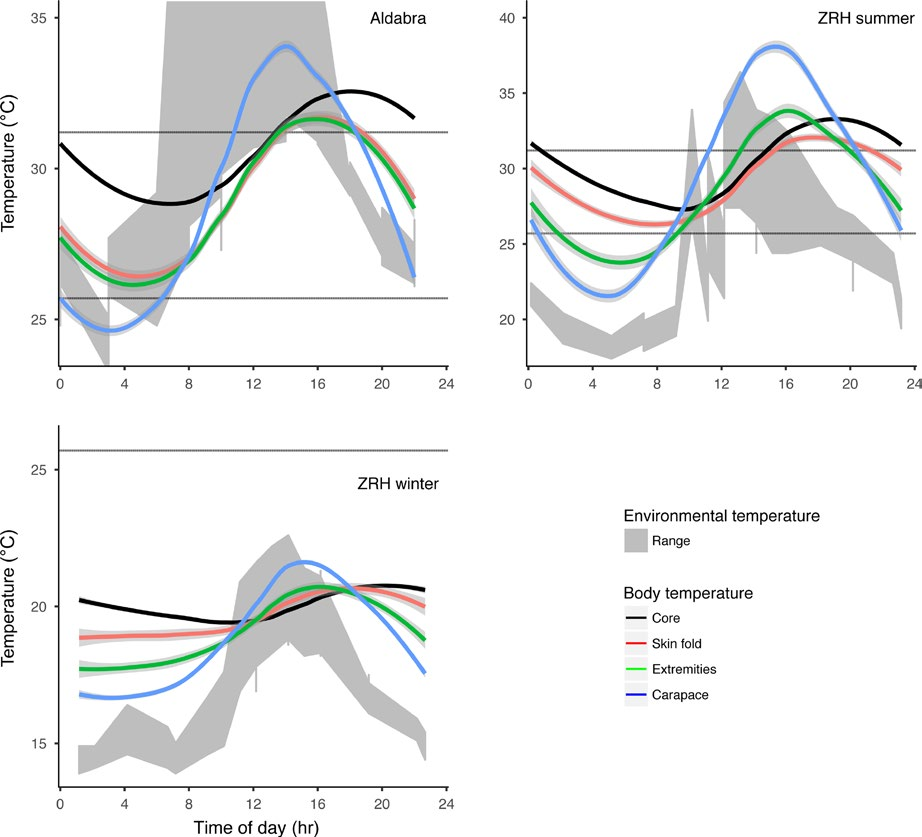
Average daily fluctuations of environmental temperature and Aldabra giant tortoise (*Aldabrachelys gigantea*) body temperatures on Aldabra Atoll and the Zurich Zoo (winter and summer). Horizontal lines depict the temperature range at which tortoise activity is maximised. Note that the *y*‐axis is scaled independently for each trial. The range intervals of *T*_*a*_ (maxima & minima) are depicted in gray shading in the background. The shading around the lines represents the 95% CI based on the line smoothing by local regression–loess. For the Aldabra trial, sun temperatures go beyond the *y*‐axis limits

There was greater variation in *T*_be_ than in *T*_bc_ (Figure 5; see S5 in Supplementary Information for summary statistics). Daily temperature fluctuations were greater on the carapace surface than on the surface of the extremities, which in turn were greater than in the skin folds or in the core body temperature. Temporal turning points of the temperature curves occurred first in the environment, followed by the carapace, the extremities, the skin folds, and, finally, the core. Temperatures of the carapace and extremities were also correlated with *T*_bc_, but to a lesser degree (*z *=* *33.38, *p *<* *.001, Kendall τ = 0.70, and *z *=* *51.28, *p *<* *.001, Kendall τ = 0.59, respectively), and remained lower than *T*_bc_ (Wilcoxon rank sum test, *p *<* *.001 in both cases). The temperature recorded in the skin folds was highly correlated with but remained lower than *T*_bc_ (*z *=* *37.77, *p *<* *.001, Kendall τ = 0.79; Wilcoxon rank sum test, *p *<* *.001).

All explanatory variables of the thermoregulation model, *trial*,* time*,* T*_air_, *mass,* and the interaction between *T*_air_ and *mass*, influenced the variation in *T*_bc_ at *p *<* *.001 (see S6 in Supplementary Information for model summary statistics). When considered independently, *T*_air_ better explained the variation in the *T*_bc_ of tortoises than mass (∆AIC = 1668) or the interaction between *T*_air_ and mass (∆AIC = 1128). The relationship between mass and *T*_bc_ in ZRH winter is bell‐shaped, increasing until it reaches 100 kg and then decreasing again. In contrast, *T*_bc_ did not exhibit any clear pattern in relation to mass in the summer trial (where no juveniles were used, and hence, the body mass range was much smaller than during winter), while on Aldabra *T*_bc_ showed a slight increase with mass (Table 2). Similar patterns to those exhibited by the relationship between mass and *T*_bc_ for each trial were observed for the acrophase, and the time lag of *T*_bc_ to acrophase (i.e. the time difference at which *T*_bc_ reaches the acrophase in relation to *T*_a_).

**Figure 6:**
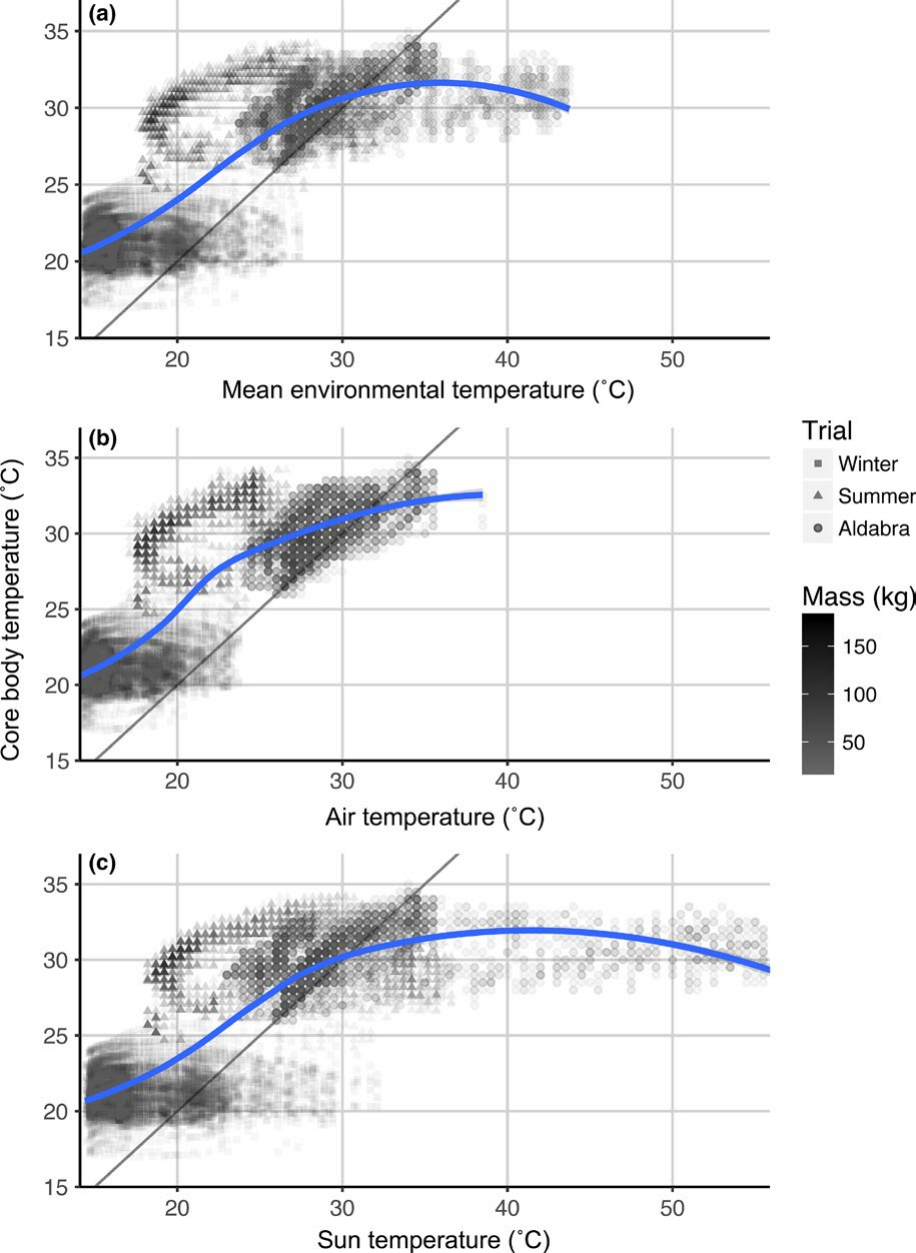
Aldabra giant tortoise (*Aldabrachelys gigantea*) core body temperature (*T*_*bc*_) in relation to mean environmental temperatures (*T*_a_), air temperature (*T*_air_, in the shade; b), and in the sun‐exposed areas (*T*_sun_; c). Points represent the *T*_*bc*_ of tortoises, with the gray‐color gradient indicating body mass, and trials indicated by shape. The solid‐line represents a 1:1 response, while the blue lines represent the response of the tortoises’ *T*_*bc*_ (all animals per trial combined) to environmental temperatures based on smoothing by local regression–loess (the shading represents the 95% CI)

For the relationship between *T*_a_ and *T*_bc_ for all tortoises in our study, most of the data point lay above *T*_a_ and slowly shifted toward lower values after *T*_a_ reached >30°C (Figure 6). As before, the variation in *T*_bc_ decreased with increasing size only during the winter trial. Interestingly, the *T*_bc_ trend increased more sharply with increasing *T*_air_ (temperature of shaded areas where they seek refuge) in contrast to *T*_sun_.

### 3.3 Body size and temperature in testudinidae

In addition to our data, we were able to gather 22 measurements of six species in four genera, from six studies (Benedict, 1932; Huot‐Daubremont, Grenot, & Bradshaw, 1996; Mackay, 1964; McMaster & Downs, 2013c; Meek & Jayes, 1982; Swingland & Frazier, 1979). Seven of these 22 data points contain estimated mass values from various individuals, and methods for measuring temperature varied (see S7 in Supplementary Information for details).

**Figure 7:**
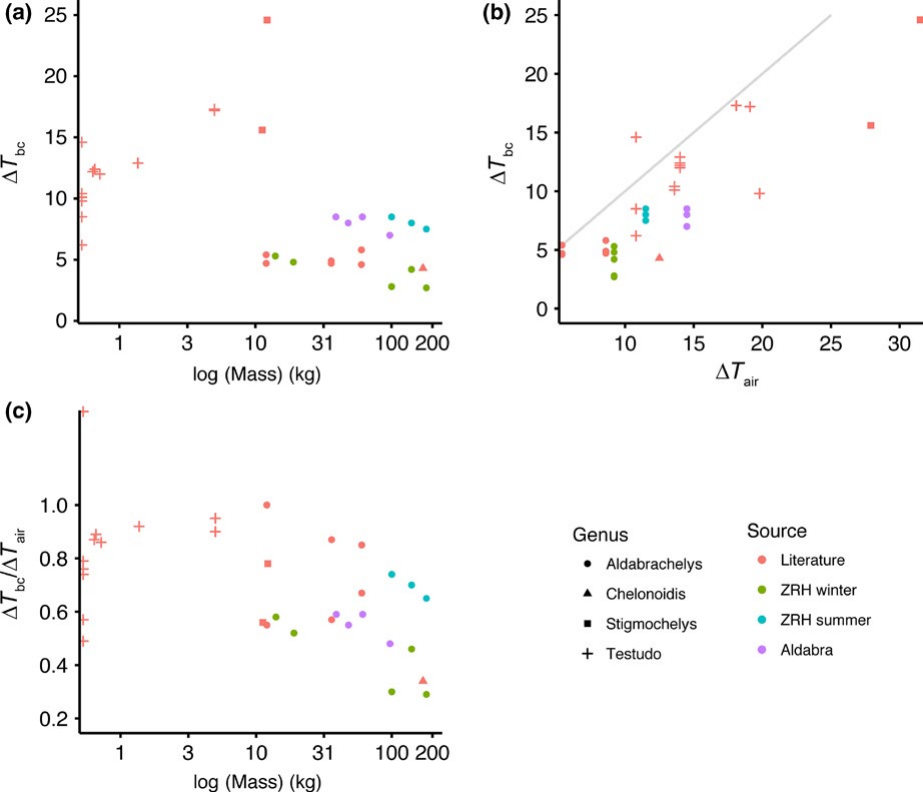
Core body temperature ranges (∆*T*_*bc*_) of Testudines in relation to the environmental temperature range (∆*T*_air_) and their mass. Relationship between ∆*T*_*bc*_ and mass (a), ∆*T*_*bc*_ and ∆*T*_*air*_ (b), and between the ∆*T*_*bc*_ and ∆*T*_air_ ratio (to control for environmental temperature; c). Point shapes indicate the genus, and color indicates the source of the data. In Panel c, one of the points is >1, probably because the air temperature did not capture the environmental temperature at which the tortoise was exposed (e.g., if the tortoise was mainly basking in the sun)

The ∆*T*_bc_ of different Testudinid species was negatively correlated with their mass (*z *=* *−3.11, *p *=* *.002, *Kendall* τ −0.38; Figure 7a). However, the ∆*T*_bc_ of tortoises was more strongly positively correlated with ∆*T*_air_ (*z *=* *4.60, *p *<* *.001, Kendall τ = 0.57; Figure 7b). When corrected for ∆*T*_air_ (using the ratio of the of ∆*T*_bc_ to ∆*T*_air_), there was a weaker negative correlation between the ∆*T*_bc_/∆*T*_air_ and *mass* (*z *=* *−2.42, *p *=* *.02, *Kendall* τ –0.29; Figure 7c).

## 4 DISCUSSION

Abiotic, biotic, physiological, and behavioral factors play an important role in the regulation of body temperature of reptiles. Here, we described the activity patterns of Aldabra giant tortoises in relation to the environmental temperature, the optimum environmental temperature at which tortoises maximize their activity, and how different gradients of environmental temperatures and body mass influenced the variation in their internal and external body temperatures. Moreover, we found evidence of thermal inertia, but this effect seemed to be context dependent in terms of the environmental temperatures experienced by the tortoises, and the overall Testudinidae dataset indicated little effect of mass on the temperature stability of tortoises.

### 4.1 Environmental temperatures and tortoise activity patterns

We used activity patterns of Aldabra tortoises in response to *T*_air_ to identify the temperature range at which activity is maximized and use this as a proxy for optimal environmental temperature range, *T*_a‐opt_. The activity of Aldabra giant tortoises is similar to that of other tortoise species, including southern Africa's largest tortoise, the leopard tortoise (*Stigmochelys pardalis*; Monadjem, McCleery, & Collier, 2013; McMaster & Downs, 2013a,2013c). Similar to Aldabra giant tortoises (R. P. Baxter et al., unpubl. data.), leopard tortoises exhibit a transition in daily activity patterns across seasons, being unimodal during the dry‐cold season, and shifting progressively toward bimodal as the season transitions toward hot and wet. In addition, under similar *T*_a_ conditions as those experienced by Aldabra giant tortoises (i.e., during the Nama‐Karoo South African summer), leopard tortoises started becoming inactive when *T*_a_ increased above 30.5–32°C between 10:00 and 11:00 hr (McMaster & Downs, 2013a), when presumably tortoises seek shade to cool down. Moreover, Lambert (1981) found similar relationships between temperature and activity in the spur‐thighed tortoise (*Testudo graeca*), although these tortoises were active at a lower temperature range of 18–28°C and inactive when *T*_a_ was below 18°C. The *T*_a_ threshold for switching from a unimodal to bimodal activity pattern in *T. graeca* was 28°C.

Aldabra giant tortoises maximize their activity (*T*_a‐opt_) in the temperature range of 25.8–31.7°C. This is within the preferred temperature range (i.e., the range of *T*_bc_ within which an ectotherm seeks to maintain itself by behavioral means) of other Testudinid species, with reported ranges of 25–31°C (*Gopherus agassizii*, Woodbury & Hardy, 1948; *G. agassizii* and *Testudo hermanni*, Brattstrom, 1965; *T. marginata* and *T. hermanni*, Panagiota & Valakos, 1992; Huot‐Daubremont et al., 1996). Moreover, the range of mean *T*_bc_ maintained by wild Galápagos (*Chelonoidis nigra*) and Aldabra giant tortoises was within *T*_a‐opt_ (Mackay, 1964; Swingland & Frazier, 1979; this study). Rather than preferred body temperatures, we calculated *T*_a‐opt_, and our environmental temperature measurements were performed in the shade. Because tortoises may be exposed to the sun when active, our measured optimal environmental temperature range may be biased to the lower limits of *T*_a‐opt_ (i.e., *T*_a‐opt_ may actually be higher). However, the pronounced drop in activity probability once temperature in the shade (*T*_air_) increases above 31°C (upper limit of *T*_a‐opt range_), as well as the clear thermoregulatory pattern exhibited by tortoises when *T*_bc_ reaches this temperature, suggests that the upper limit of the preferred core body temperature range is around 31°C. This is in accordance with the findings of Swingland and Frazier (1979), who reported the maximum critical temperature for Aldabra giant tortoises in the wild to be 36–38°C (measured in tortoises dying or that recently died from overheating in the wild). Therefore, the *T*_a‐opt range_ in combination with the mean *T*_bc_ of wild tortoises may serve as a reference for a high quality thermal environment for husbandry and captive care practices (e.g., McArthur & Barrows, 2008) and conservation efforts with regard to climate change and thermal refuge availability (e.g., Moulherat et al., 2014; Swingland & Frazier, 1979).

Although the activity of tortoises was affected by seasonality, our results are likely to capture the full extent of environmental temperatures at which Aldabra tortoises maximize their activity because we include activity and temperature data from a 2‐year period. However, the data show the flexibility of tortoises with regard to their activity patterns and available *T*_air_, depicted by the variation in the seasonal activity patterns in relation to temperature. Other tortoises also show flexibility in terms of their activity in relation to seasonal changes in temperature (among other factors, see McMaster & Downs, 2013a). Rainfall and temperature have been shown to be important factors influencing the activity of tortoises (Kazmaier, Hellgren, & Synatzske, 2011). In our activity model, the interaction between the dry season and *T*_air_ negatively affected activity. We thus hypothesize that the changes in activity patterns dependent on seasons in Aldabra giant tortoises may also be driven by changes in water balance and water conservation. Water balance is important in tortoises because it affects their food consumption, diet, daily behavior, osmoregulation, and body mass (Nagy & Medica, 1986). Tortoises have been shown to suffer significant evaporative water loss from their integuments and through respiration (Schmidt‐Nielse & Bentley, 1966). Moreover, increasing temperatures and drought conditions have been shown to increase water loss through evaporation in tortoises (Cloudsley‐Thompson, 1968; Minnich, 1977). The expected main mechanism for thermoregulation in tortoises is to change their daily activity levels and behavior (because the rigid shell limits the effectiveness of postural changes during behavioral thermoregulation; McMaster & Downs, 2013c). To conserve water, tortoises may decrease their activity levels and stay in the shade during and after the warmest part of the day in the dry season. If our hypothesis is correct, we can expect that the increasing frequency of drought periods on Aldabra (Haverkamp et al., 2017) will have negative impacts on the activity of giant tortoises.

It should be noted that temperature is not the only factor influencing the activity of tortoises. For example, Gibson and Hamilton (1983) hypothesized that seasonal changes in the activity of Aldabra giant tortoises were in response to food availability. Moreover, precipitation, solar radiation, and wind velocity also seem to play a role in determining the bimodal to unimodal activity patterns and the activity levels of Aldabra giant tortoises (unpubl. data). Further research is being undertaken to determine the environmental drivers of the activity of Aldabra giant tortoises on Aldabra.

### 4.2 Body temperature of *Aldabrachelys gigantea*

Fluctuations in *T*_bc_ lagged behind those of *T*_a_, and in general tortoises heated more rapidly during the day than they lost heat during the night, when *T*_bc_ fell slowly. During the ZRH winter trial, *T*_bc_ was always higher than *T*_air_; only the temperature logger placed in direct sunlight recorded temperatures that were higher than *T*_bc_, especially during the middle of the day. *T*_air_ better explained *T*_bc_ of tortoises, and in general, they were able to maintain their *T*_bc_ above low *T*_air_, and below high *T*_air_, and their *T*_bc_ was affected by the range of available environmental temperatures. Moreover, the temperatures measured on the surface of the carapaces were notably higher than *T*_air_ during the morning, which is evidence for thermoregulation via basking behavior (e.g., Crawford, Spotila, & Standora, 1983; Lambert, 1981). While basking, reptiles reach higher core temperatures than air temperatures, and *T*_bc_ correlates positively with time spent basking (Boyer, 1965; Rivera‐Vélez & Lewis, 1994). Correspondingly, *T*_bc_ dropped in cloudy days, when basking was not possible, and environmental temperatures dropped by 3–5°C. During ZRH summer and on Aldabra, the mean and the range of *T*_a_ were higher, and tortoises were able to maintain their *T*_bc_ close to *T*_a‐opt_. On Aldabra, we recorded some extreme high temperatures in the sun during the second half of the study, probably due to clear skies and virtually no wind, but tortoises were able to maintain a stable *T*_bc_ nonetheless. Swingland and Frazier (1979) reported very similar patterns of *T*_bc_ for *A. gigantea* to those exhibited by our tortoises, but in the southeast of the atoll on Grande Terre Island, where shade is more limited and where the size dimorphism of tortoises is much less pronounced. Additionally, Mackay (1964) observed similar patterns of *T*_bc_ in two Galápagos giant tortoises (65 and 170 kg), which were able to maintain their *T*_bc_ within ~28–32°C when the mean *T*_a_ was ca. 28°C and fluctuated between ca. 23–35°C, despite the difference in their mass.

As found in leopard tortoises (McMaster & Downs, 2013b), the differential variation in *T*_be_ of different body surfaces and as well as that of *T*_bc_ indicated that there are large thermal gradients within the bodies of Aldabra giant tortoises. For example, the maximum carapace temperature of Aldabra giant tortoises in our study sometimes greatly exceeded that of their recorded *T*_bc_, to the point that it surpassed the maximum critical temperature (of *T*_bc_) recorded for the species. Studying the thermoregulation of Galápagos giant tortoises, Mackay (1964) proposed, based on the temperature differential between the core body temperature and the carapace, that heat flows through a limited region with high resistance when compared to that of the material absorbing the heat. We found the same pattern in Aldabra giant tortoises, where the integral of the difference between the *T*_bc_ and the carapace temperature (mean of the vertebral and costal scutes) followed the same temporal pattern as that of *T*_bc_. While in the shade, the temperature of the scutes on the carapace of the tortoises remained above shaded environmental temperatures as well as above the *T*_bc_, indicating that they lost heat to the environment. In contrast, at night, carapace temperatures dropped below *T*_bc_ and were closer to *T*_a_ than that of the extremities or skin folds (which were also below *T*_bc_). Thus, our results suggest that Aldabra giant tortoises employ different behavioral and physiological mechanisms to use their carapace as a heat exchanger: a heat collector in the mornings, a radiator (i.e., cooling system) during the warmest part of the day, and an insulator during the coldest part (see McMaster & Downs, 2013b, and references therein, for a discussion of the differential temperature of *T*_bc_ and *T*_be_ in tortoises and possible control mechanisms).

It is worth noting that Aldabra hosted a population of introduced goats until 2012, when an eradication program was completed (Bunbury et al., 2013). In 1985, during the same time as the tortoise population decline from an estimated 130,000 to around 100,000 (Bourn et al., 1999), Coblentz and Vuren (1987) estimated that there were as many as 1,300 goats on Aldabra. They suggested that the major impact of the goats was their negative effect on shade resources through overbrowsing, rather than direct competition with tortoises for food. Our results suggest a strong role of available shade for structuring tortoise activity and in body temperature regulation, supporting the likelihood of overbrowsing by goats having had a negative impact on Aldabra's giant tortoises.

### 4.3 Body size and temperature in testudinidae

Thermal inertia likely explains why after basking, tortoises at the Zurich Zoo had higher *T*_bc_ than minimum *T*_a_ during early mornings on the next day, and cooling rates appeared to decrease with size. However, the effect of mass on temperature stability of Aldabra giant tortoises (∆*T*_bc_) differed by trial. When the mean *T*_a_ was 17.2°C during the ZRH winter trial, *T*_bc_ stability increased with increasing size. This was not the case for the mean *T*_bc_. In ZRH winter, the tortoise of intermediate size (100 kg) had the highest mean *T*_bc_, suggesting that both the fast cooling rate of small animals and the slow heating rate of larger animals influenced their mean *T*_bc_. On the other hand, ∆*T*_bc_, as well as the mean core body temperatures in ZRH summer and on Aldabra, did not seem to vary with mass. Furthermore, there was a tendency for the acrophase of tortoises (time at which *T*_bc_ peaked), and of the thermal lag of *T*_bc_ to *T*_a_, to increase with mass in the Zurich tortoises, but there was no apparent trend on Aldabra. Also, the effect size of mass in our thermoregulation model was rather small, and *T*_air_ better explained changes in the *T*_bc_ of Aldabra giant tortoises. Moreover, the analysis on the overall Testudinidae dataset appears to indicate that within the body size range of the tortoise species studied, large individuals are subject to similar fluctuations in body temperature as smaller ones once variation in air temperature has been taken into account. Thus, the presence of thermal inertia in tortoises seems to depend on the environmental temperatures. However, it is notable that behavioral thermoregulation and acclimatization can potentially override the effects of mass on the *T*_bc_ of tortoises and thus provide an alternative and possibly synergistic explanation of the apparent context‐dependent effect of mass on *T*_bc_.

In addition, the notion that large ectotherms may maintain a high (30°C) and stable *T*_bc_ within a narrow range (2°C) due to mass‐dependent thermal inertia, similar to homeothermic endotherms, is often referred to as “inertial homeothermy” (McNab & Auffenberg, 1976; Seebacher, 2003). Despite being “giants,” even under stable conditions, the range of *T*_bc_ in Aldabra giant tortoises (as well as other species of smaller tortoises) was much larger than 2°C. These results indicate that that inertial homeothermy is not possible in tortoises with the range of body masses studied. These findings support the conclusion of Grigg, Beard, and Augee (2004), who found that inertial homeothermy over the course of a single day is only found in large ectotherms above 500 kg of body size.

## 5 CONCLUSIONS

Despite the large sizes that Aldabra giant tortoises can attain, and the presence of thermal inertia, our results suggest that tortoises are incapable of regulating their core body temperatures within a range narrow enough to be considered inertial homeotherms. Rather, the interplay between the mass of the tortoises and the variation of *T*_a_, in combination with behavioral thermoregulation, limits the degree to which these ectotherms can attain core body temperatures close to their presumed optimum *T*_bc_. We found evidence of thermoregulation, where tortoises were able to maintain *T*_bc_s independent of *T*_a_. We also found instances of thermoconformity, and when we evaluated *T*_bc_ in response to *T*_a_, our results indicate that giant tortoises act as mixed conformer–regulators (Willmer et al., 2005). However, although the relationship between *T*_bc_ and *T*_a_ suggests that giant tortoises can maintain a stable *T*_bc_ when the mean *T*_a_ is above the lethal temperature (>36°C), evaluating the components of *T*_a_ independently (the temperature of shade and sun‐exposed loggers) shows that Aldabra giant tortoises have a limit: when shade temperature (*T*_air_) surpasses ca. 31°C, the *T*_bc_ seems to keep increasing rather than reaching a plateau. While some tortoises can adjust their behavior to survive extreme environmental temperatures (e.g., *Gopherus agassizii* can remain active even when *T*_a_ reaches ca. 60°C by adjusting the time spent in burrows; Zimmerman et al., 1994), larger animals such as *A. gigantea* have limited options. The plasticity and intraspecific and interspecific variation in tortoises, and other reptiles, certainly allow for the animals to respond to the selective pressures imposed by the environment. However, it is likely that climate change will accentuate thermoregulatory pressures (Barrows, 2011; Gunderson & Stillman, 2015), especially on larger species.

## DATA ACCESSIBILITY

The data used for this article are accessible through Dryad (http://datadryad.org/resource/doi:10.5061/dryad.b4v22).

## CONFLICT OF INTEREST

None declared.

## AUTHORS’ CONTRIBUTIONS

WF, RB, DH designed the Aldabra part of this study and collected the data and SF, MB, JMH, MC designed the Zurich part of this study; SF and MC collected the data. WF, MC, DH, and AO analyzed the data; WF, MC, DH performed the literature review and drafted a version of the manuscript that then received input from all coauthors.
