# Supplementary material for: Patterns of activity and body temperature of Aldabra giant tortoises in relation to environmental temperature
Source: Ecol Evol. 2018 Jan 19;8(4):2108–21. doi: 10.1002/ece3.3766 (PMC5817133; doi:10.1002/ece3.3766)
Supplement: Supplementary file 1 [file ECE3-8-2108-s001.pdf]

Supporting Online Information

S1: Time discretisation for the activity periods of Aldabra giant tortoises (*Aldabrachelys gigantea*) on Aldabra Atoll to account for the non-linear relationship between time and activity. We discretised continuous time into four periods (I– IV), following the overall activity turning points through time, and comprising 06:00–08:00, 08:15–13:30, 13:30–17:30 and 17:45–20:00, respectively

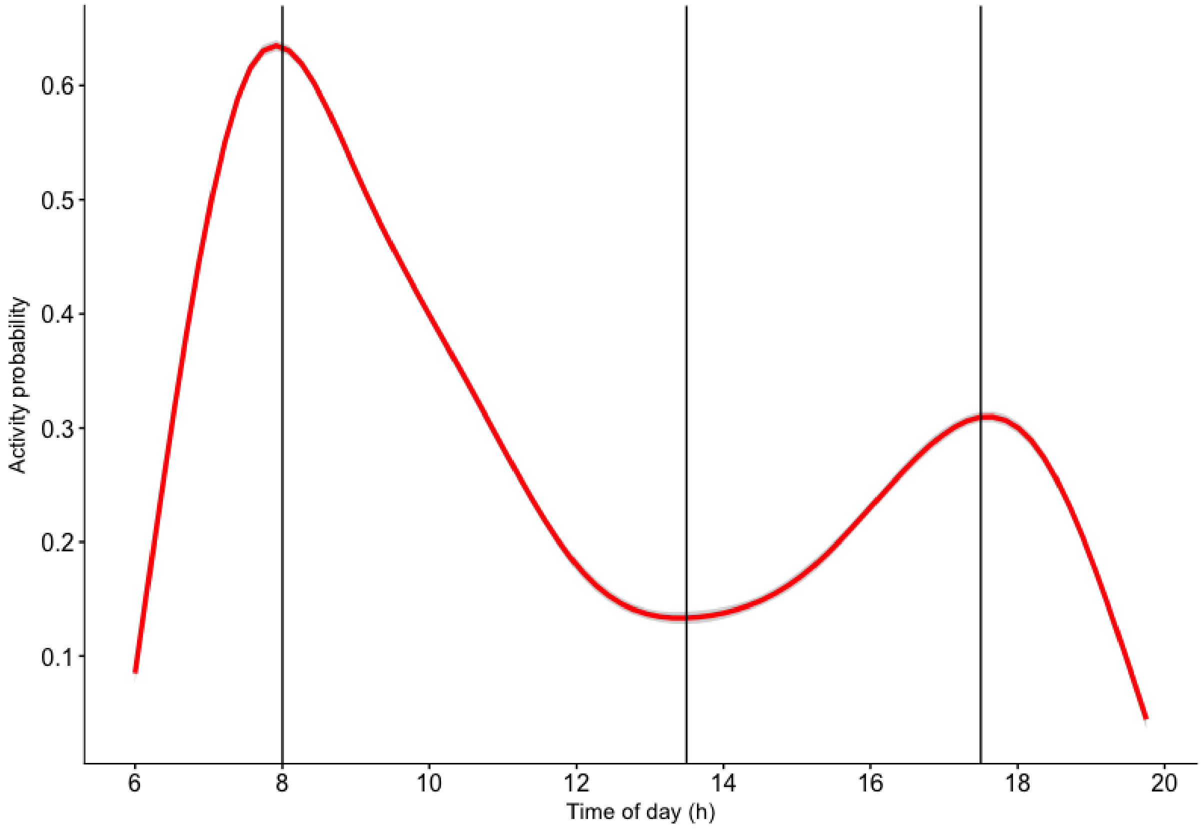

S2: Time discretisation for the thermoregulation periods of Aldabra giant tortoises (*Aldabrachelys gigantea*) in each trial to account for the non-linear relationship between time and core body temperature (T_{bc}). Continuous time was discretised into three periods (I, II, and III) comprising the morning period when tortoises are cooling down, the morning-afternoon period when tortoises are heating up, and the night period when tortoises start to cool down, respectively, for each trial independently. Lines depict the Tbc of each individual of a given mass (legend) per trial, with the 95% CI by local regression, loess.

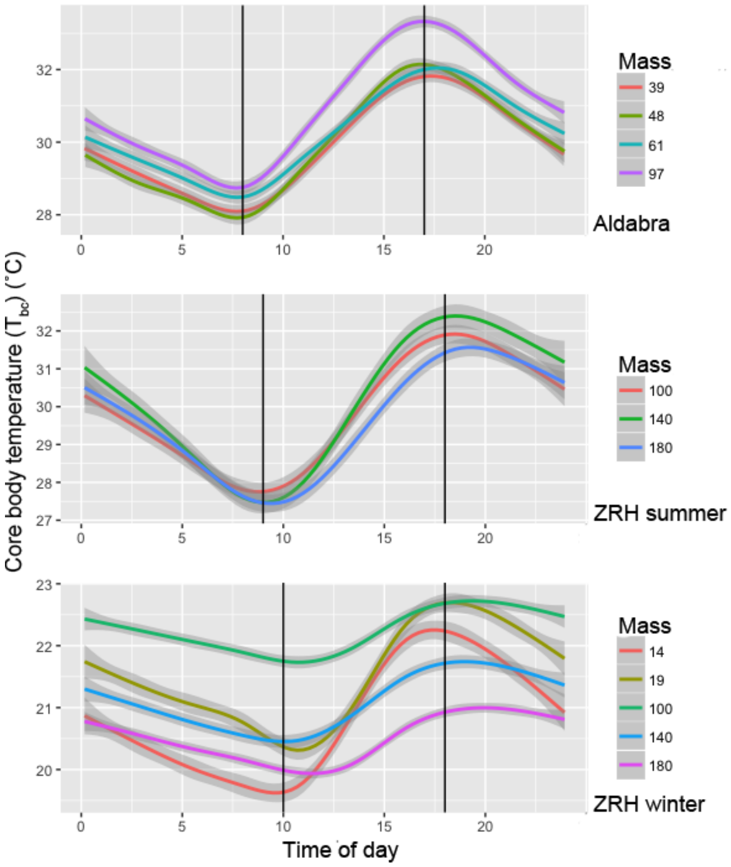

S3: Results of the mixed-effects model on the influence of air temperatures on the activity of Aldabra giant tortoises on Aldabra Atoll.

Fixed effect	Estimate	SE	*p*-value
Intercept	0.87	0.30	0.003
$year_{II}$	0.01	0.05	0.89
$time_{II}$	10.03	0.24	< 0.001
$time_{III}$	3.74	0.26	< 0.001
$time_{IV}$	-10.22	0.28	< 0.001
T_{air}	-0.04	0.01	< 0.001
$season_{dry}$	3.09	0.16	< 0.001
$time_{II}$: T_{air}	-0.34	0.01	< 0.001
$time_{III}$: T_{air}	-0.15	0.01	< 0.001
$time_{IV}$: T_{air}	0.33	0.01	< 0.001
$season_{dry}$: T_{air}	-0.13	0.01	< 0.001

S4: Activity seasonality of giant tortoises (*Aldabrachelys gigantea*) on Aldabra Atoll in relation to air temperature. Vertical lines depict the air temperature range at which activity is maximised when the two seasons are combined.

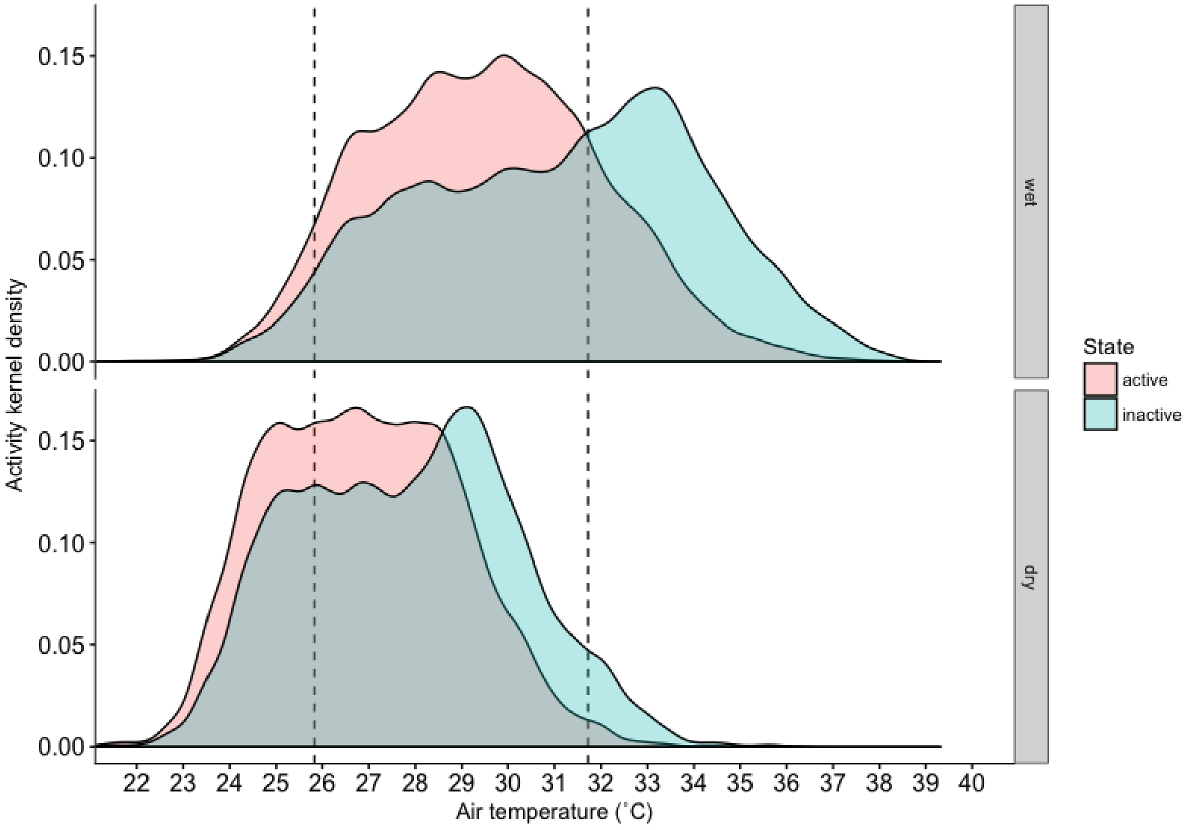

S5: Summary statistics of the external body temperatures (T_{be}) of Aldabra giant tortoises (*Aldabrachelys gigantea*), with different size and body mass, exposed to different thermal environments.

Skin folds

Tortoise (mass)	Study	Mean	Min	Max	Range	±SD
JVS (14kg)	ZRH Winter	18.5	16.1	21.4	5.3	1.5
JVL (19kg)	ZRH Winter	19.8	17.1	21.5	4.4	1.0
HMA (100kg)	ZRH Winter	20.7	18.7	22.0	3.3	0.8
SBY (140kg)	ZRH Winter	19.7	18.6	21.1	2.5	0.6
BBY (180kg)	ZRH Winter	19.8	18.3	21.3	3.0	0.7
HMA (100kg)	ZRH Summer	29.6	25.2	33.4	8.2	2.5
SBY (140kg)	ZRH Summer	29.2	25.5	33.4	7.9	2.3
BBY (180kg)	ZRH Summer	28.5	24.2	33.0	8.8	2.0
BEL (39kg)	Aldabra	28.6	25.0	33.0	8.0	2.1
WIL (35kg)	Aldabra	29.7	23.7	34.0	10.3	2.6
UNM (48kg)	Aldabra	28.7	24.2	33.5	9.3	2.2
CFK (61kg)	Aldabra	28.8	24.9	33.5	8.6	2.2
LDX (97kg)	Aldabra	29.6	25.6	33.7	8.1	2.0

Extremities

Tortoise (mass)	Study	Mean	Min	Max	Range	±SD
JVS (14kg)	ZRH Winter	18.3	15.4	22.0	6.6	1.8
JVL (19kg)	ZRH Winter	19.4	16.8	21.9	5.1	1.3
HMA (100kg)	ZRH Winter	20.0	17.2	21.9	4.7	1.3
SBY (140kg)	ZRH Winter	19.2	17.3	21.0	3.7	0.9
BBY (180kg)	ZRH Winter	19.4	16.6	21.4	4.8	1.1
HMA (100kg)	ZRH Summer	29.3	21.9	37.4	15.5	3.7
SBY (140kg)	ZRH Summer	29.1	23.0	37.8	14.8	3.6
BBY (180kg)	ZRH Summer	28.5	22.8	37.7	14.9	3.3
BEL (39kg)	Aldabra	28.7	24.7	33.3	8.6	2.1
WIL (35kg)	Aldabra	29.4	21.4	34.4	13.0	2.9
UNM (48kg)	Aldabra	28.5	22.5	33.5	11.0	2.4
CFK (61kg)	Aldabra	28.7	24.4	33.5	9.1	2.3
LDX (97kg)	Aldabra	29.5	25.6	33.5	7.9	2.1

Carapace

Tortoise (mass)	Study	Mean	Min	Max	Range	±SD
JVS (14kg)	ZRH Winter	18.6	14.7	23.2	8.5	2.2
JVL (19kg)	ZRH Winter	19.5	16.0	22.3	6.3	1.9
HMA (100kg)	ZRH Winter	19.5	15.8	22.4	6.6	1.9
SBY (140kg)	ZRH Winter	19.1	15.6	23.2	7.6	1.8
BBY (180kg)	ZRH Winter	19.2	15.4	22.7	7.3	2.0

HMA (100kg)	ZRH Summer	30.8	19.8	44.2	24.4	6.5
SBY (140kg)	ZRH Summer	30.2	19.8	42.7	22.9	6.4
BBY (180kg)	ZRH Summer	29.9	20.6	41.9	21.3	6.1
BEL (39kg)	Aldabra	28.9	22.9	38.5	15.6	3.2
WIL (35kg)	Aldabra	28.9	20.6	41.6	21.0	4.5
UNM (48kg)	Aldabra	28.1	20.6	37.3	16.7	3.7
CFK (61kg)	Aldabra	28.9	22.9	38.3	15.4	3.4
LDX (97kg)	Aldabra	29.9	24.1	47.1	23.0	4.1

S6: Results of the mixed effects model of Aldabra giant tortoise core body temperature.

**Fixed effects**	**Estimate**	**SE**	***p*-value**
Intercept	14.79	0.50	<0.001
$Trial_{ZRH\ summer}$	7.91	0.57	<0.001
$Trial_{Aldabra}$	5.73	0.51	<0.001
$time_{II}$	-0.26	0.03	<0.001
$time_{III}$	1.32	0.02	<0.001
T_{air}	0.36	0.01	<0.001
mass	0.03	0.00	<0.001
T_{air}:*mass*	0.00	0.00	<0.001

S7: Details for the used for the literature review on Testudinidae core body temperature in relation to air temperature and mass (**Excel file**).
